# HER2-Positive Inflammatory Breast Cancer Challenges of Clinical Practices

**DOI:** 10.7759/cureus.22925

**Published:** 2022-03-07

**Authors:** Denise Magalhães, Inês Rangel, Alexandra Mesquita

**Affiliations:** 1 Oncology, Hospital Pedro Hispano, Matosinhos, PRT; 2 Cardiology, Hospital Pedro Hispano, Matosinhos, PRT

**Keywords:** targeted therapy, cardiotoxicity, brain metastasis, inflammatory, breast cancer

## Abstract

HER2-positive inflammatory breast cancer (IBC) is associated with poor overall survival. Targeted therapies have led to improved outcomes. IBC is underrepresented in clinical trials due to its rareness. This case reports a 52-year-old woman diagnosed with IBC of 119x89mm, axillar node-positive, hormone receptor-negative, HER2 positive. The patient underwent neoadjuvant chemotherapy with dual HER2 blockage. Mastectomy histology showed pathological complete response. After two cycles of adjuvant trastuzumab, the patient developed asymptomatic cardiotoxicity leading to the therapeutic suspension. Early recurrence and persisting cardiac alterations prevented treatment with anti-HER2 therapy. At the time of brain recurrence, with cardio-oncology collaboration, it was possible to start TDM-1, with a reduction of 71% of brain lesions size, after two cycles. This case highlights the effectiveness of anti-HER therapy in IBC and the importance of multidisciplinary discussion in treatment choice and toxicity management.

## Introduction

Inflammatory breast cancer (IBC) accounts for 1% to 6% of all breast cancers, being the median overall survival (OS) 3.5-years shorter than non-IBC [1,2]. Early and intensive treatment is associated with improved prognosis as well as the addition of targeted therapies such as anti-HER2 [3]. Nevertheless, adverse events (AE) like cardiotoxicity can lead to therapeutic limitation.

The authors report a case of amplified HER2 IBC, with a complete response (CR) to neoadjuvant chemotherapy, followed by an early recurrence. AE leads to early therapeutic discontinuation with the need for multidisciplinary management.

## Case presentation

A 52-year-old woman, current smoker, without a family history of cancer, presented swelling and flushing in the upper quadrant of the left breast after a one-and-half month of evolution. Clinical examination revealed a breast with hard swelling, inflammatory signs, and thickened skin. Breast ultrasound showed a large mass (119x89mm) in the left breast and suspicious homolateral axillary adenopathies that revealed an invasive carcinoma, grade 3, hormonal receptors (HR) negative, HER2 positive [3+ by immunohistochemistry (IHC)], Ki67 >50%, in pathology examination - stage cT4N+M0. The patient was proposed for neoadjuvant chemotherapy - four cycles of doxorubicin (60 mg/m2), cyclophosphamide (600 mg/m2) followed by four cycles of docetaxel (75 mg/ m2), trastuzumab (600mg), pertuzumab (840 mg loading dose, followed by 420 mg). Baseline echocardiogram showed a normal systolic and diastolic function, with a left ventricular ejection fraction (LVEF) of 68%.

After completing the neoadjuvant chemotherapy scheme, the patient underwent left-side radical mastectomy and level I/II axillary dissection. Pathology examination revealed a CR. The patient was proposed to adjuvant trastuzumab (600 mg) and radiotherapy. After two cycles of adjuvant trastuzumab, the patient developed asymptomatic inferior wall akinesia, with decreased left ventricular ejection fraction (LVEF: 48%). Trastuzumab was withheld, and cardioprotective therapy started (carvedilol 6.25mg/twice daily and lisinopril 20mg/daily). Ischemic aetiology was ruled out, and cardiotoxicity was attributed to trastuzumab. The patient completed 50 Gy in 25 fractions of adjuvant radiotherapy.

Five months after trastuzumab suspension, the disease recurred locally, with the same IHC characteristics. At that point, the echocardiogram revealed a severe hypokinesis of the inferior wall, so, due to sustained cardiotoxicity, the patient was proposed to weekly paclitaxel (80mg/m2), with clinical CR.

**Figure 1:**
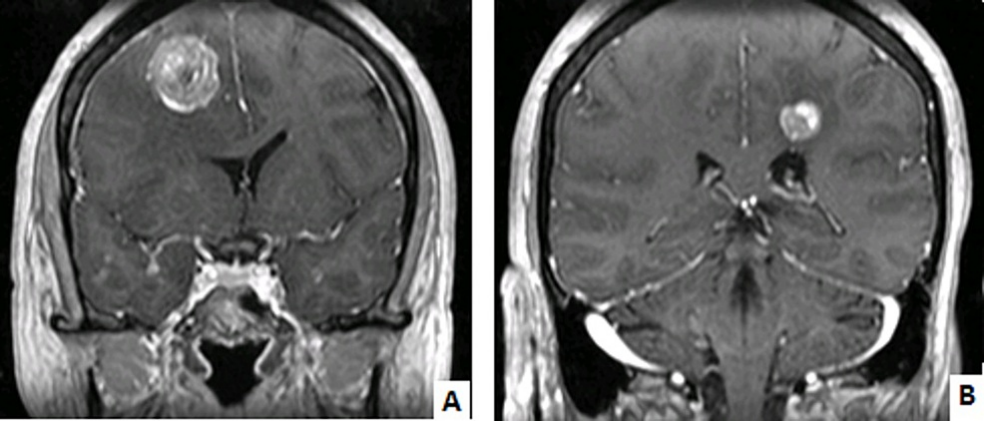
Cranial Magnetic Resonance imaging at brain recurrence A: Right frontal lobe lesion with 29 x 24 mm, oedema, and midline deviation. B: Left front parietal lobe lesion with 16 x 14 mm.

After 12 months of weekly paclitaxel, the patient presented with an unbalanced gait with fall and confusion. Cranial Computed Tomography (CT) scan and Cranial Magnetic Resonance (MR) revealed two lesions - in the right frontal lobe (29 x 24 mm) and the left front parietal lobe (16 x 14 mm) - surrounded by vasogenic edema (Figure 1).

**Figure 2:**
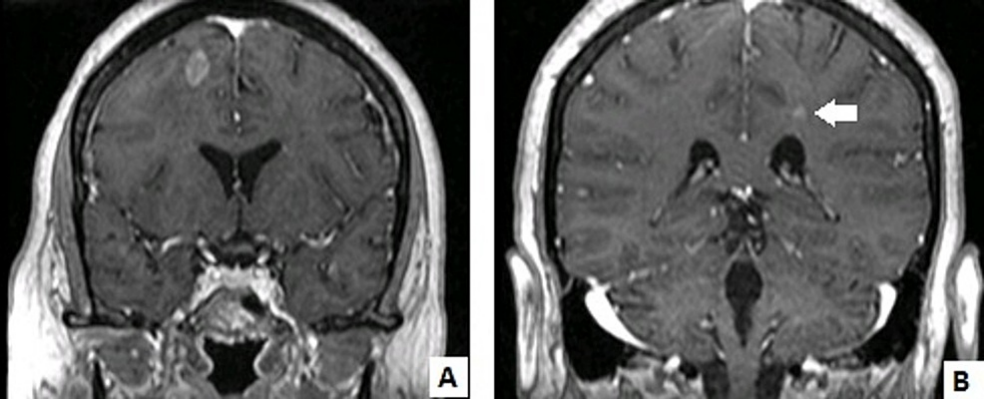
Cranial Magnetic Resonance imaging after two cycles of TDM-1. A: Right frontal lobe lesion with 9x8 mm.  B/Arrow: Left front parietal lobe lesion with 4 mm.

The patient started dexamethasone 16 mg/day, with slight symptom improvement. An echocardiogram, 20 months after trastuzumab discontinuation, showed recovery of inferior wall motion and a normal LVEF. The clinical case was discussed at the tumor board; taking into account the cardiac recovery and the metastatic setting, the patient was proposed to trastuzumab emtansine (T-DM1, 3.6 mg/Kg every three weeks), under stricter cardiac evaluation, and to brain stereotactic radiotherapy. After two cycles of T-DM1, the patient was asymptomatic, and no lesions were seen on the radiotherapy planning CT scan. Cranial MR showed a reduction in the size of the lesions (9x8 mm and 4 mm) and in the vasogenic edema (Figure 2), so it was decided not to undergo radiotherapy given the good response to TDM-1 therapy.

Currently, the patient is asymptomatic and has no evidence of cardiac events related to T-DM1, being integrated into the cardio-oncology follow-up board. Current progression-free survival (PFS) of 12 months and an OS of 41 months. 

## Discussion

The authors presented a case of an IBC, HER2 positive, with pathological complete response (pCR) to the neoadjuvant treatment. Despite many improvements in cancer treatment and outcomes, IBC is characterized by aggressive behaviour with the poorer OS when compared to non-IBC [2]. As for the subtype, it is often classified as basal-like, or HER2 enriched [4]; however, there is not enough data to correlate the IBC outcome according to HR and HER2 status [5]. Çakar et al., in a retrospective analysis, in concordance with non-IBC, identified a better outcome for the Luminal A subtype and a worse outcome for the triple-negative breast cancer (TNBC); however, Masuda et al. found no correlation between HR or HER2 positive IBC and a better outcome [5,6]. Additionally, its rareness makes it underrepresented in clinical trials, not allowing conclusions to be drawn for treatments and prognostic factors.

In the international guidelines, the combined treatment strategy - neoadjuvant chemotherapy followed by surgery and adjuvant RT - is recommended and was associated with an improved outcome [1]. Five-year disease-free survival (DFS) ranges from 22% to 48% and the median OS from 25 to 70 months with the combined-modality [3]. With the advent of targeted therapy, HER2-positive breast cancer showed an improved response rate, with an increase of 16% in pCR with neoadjuvant HER2 dual blockade [1,7]. Although the NeoSphere trial included patients with IBC, this accounted for only 7% of the population, precluding a subgroup analysis [7]. In a retrospective analysis of patients with IBC being treated in MD Anderson, the efficacy of anti-HER2 therapy in terms of prolonging OS and DFS did not reach statistical significance, despite improving pCR [6]. Thus, the real benefit of anti-HER2 therapy and dual blockade in IBC is not completely known. The case described shows the benefit of HER2 targeted therapy in IBC not only in the early setting, with a pCR, but also in the advanced stage.

pCR was defined as a surrogate endpoint in the early stage, particularly in HER2 positive/HR negative and TNBC, notwithstanding that our patient presented an early recurrence. In the NeoSphere trial, at the five-year follow-up, a correlation between pCR and PFS was made. The five-year PFS was 85% in the patients achieving pCR, versus 76% in non-pCR [8]. Additionally, Masuda et al. reported an association between pCR and longer OS in the HER-2 positive/HR negative and TNBC subtypes in IBC [6]. Therefore, it remains unclear whether this early recurrence was due to IBC nature or if it was related to premature adjuvant trastuzumab withhold due to cardiotoxicity.

Cardiotoxicity is one of many challenges faced with HER2 targeted therapy that narrows its use and benefit in clinical practice. In the NeoSphere trial, 18 patients discontinued treatment due to study drug-related AE - six patients due to cardiac events [7]. Cardiac dysfunction related to adjuvant trastuzumab is described in 5% to 10% of patients in the clinical trials, most with recovery without any intervention [7]. In the case described here, the patient’s development of asymptomatic inferior wall akinesia and decreased LVEF was attributed to trastuzumab therapy. The patient started cardioprotective therapy with the intent of resuming trastuzumab; however, the toxicity was sustained. The underlying mechanism of trastuzumab-induced cardiotoxicity has been investigated - neuregulin-1 binds to HER2, initiating a signaling pathway essential to cardiomyocytes survival when HER2 is targeted mitochondrial dysfunction occurs, which promotes apoptosis and oxidative stress. Furthermore, there is a reduction in transcription factors essential to cell survival [9]. Some conditions were identified as risk factors for trastuzumab-related cardiotoxicities, such as advanced age, hypertension, diabetes, previous anthracycline use, and low baseline LVEF [10]. Radiotherapy to the left chest wall was also investigated as a risk factor; however, the findings were contradictory, and most studies point towards the absence of correlation [10]. The only established risk factor the patient presented was previous treatment with an anthracycline. Additionally, many studies have addressed the benefit of primary prevention with neurohormonal antagonist medications. Even though the data is inconsistent, it appears to be a benefit in patients with a high risk of cardiotoxicity, as patients were previously exposed to anthracycline [11].

Another challenge faced in HER2-positive tumors is the high frequency of brain metastasis, without benefit in adjuvant targeted therapy in precluding brain recurrence [12]. The presence of brain metastases is associated with poor outcomes [13]. Brain metastases are managed with local therapies such as surgery, stereotactic radiotherapy, or whole-brain radiotherapy and with systemic therapy [12]. Some systemic targeted therapy has demonstrated effectiveness, such as Lapatinib or Neratinib in combination with Capecitabine, Tucatinib-Capecitabine-Trastuzumab, T-DM1, and more recently Trastuzumab deruxtecan (T-Dxd) [13-18].

In an exploratory analysis from the KAMILLA trial, a ≥30% reduction in the sum of the largest diameters of target brain lesions was observed in 49.3% of the patients with T-DM1 in the absence of brain radiotherapy; and a PFS of five months [13]. In the HER2CLIMB trial, the median intracranial objective response rate was 47%, with a median duration of intracranial response of 6.8 months [17]. In a subgroup analysis of patients with brain metastases of the DESTINY-Breast01 trial, the objective response rate and the median PFS were 58% and 18 months, respectively [18]. DESTINY-Breast03, in the post hoc analysis, demonstrated an improvement in PFS from 5.7 months with T-DM1 to 15 months with T-Dxd towards brain metastases [19]. These trials evidence the benefit of targeted therapy in brain metastases; even though no trial has been conducted in the IBC subtype, our patient benefited from T-DM1 with a 71% reduction in brain lesions after just two cycles. The improvement in symptoms allowed corticotherapy discontinuation.

It is also important to highlight the PFS of 12 months and the pCR with weekly paclitaxel, being the median PFS reported in clinical trials of nine months [20]. From our investigation, there are no reported factors associated with a good response to taxanes in IBC.

The challenge of this clinical case and the absence of prospective data to support these clinical decisions emphasize the need for multidisciplinary discussion. The integration of medical specialties in a multidisciplinary analysis allows a better approach to therapy-related toxicities, improving disease treatment and patients’ quality of life. This collaboration had a preponderant role in the major outcome of our patients. Emphasizing the inflammatory aetiology, it becomes crucial to create clinical trials to define the best systemic therapy and improve the poor outcome.

## Conclusions

IBC is associated with poor outcomes; its rareness and the absence of prospective clinical trials preclude specific treatments progression. Thus, therapy guidelines result from an extrapolation of non-IBC. The evolution of HER2-targeted therapy is associated with an improvement in clinical outcome, although the benefit of each drug is not known in the inflammatory subtype. Clinical trials are urgently needed as well as the reports of clinical practice. In addition, the multidisciplinary discussion is crucial to the management of these patients and clinical challenges.
